# Which Outcome in Chronic Kidney Disease-Mineral and Bone Disorder Patients?

**DOI:** 10.5812/numonthly.18662

**Published:** 2014-04-28

**Authors:** Mario Cozzolino

**Affiliations:** 1 Laboratory of Experimental Nephrology, Renal Division, DiSS University of Milan, San Paolo Hospital, Milan, Italy

**Keywords:** Dialysis, Renal Insufficiency, Chronic, Quality of Life

Dear Editor,

Secondary hyperparathyroidism (SHPT) is one the major complications in dialysis patients. Hyperphosphatemia, hypocalcemia, low serum vitamin D levels are greatly involved into the physiopathology of SHPT in chronic kidney disease (CKD), in which the down-regulation of parathyroid Vitamin D Receptor (VDR) and Calcium Sensing Receptor (CaSR) represent a critical step. Moreover, new data demonstrate that the Fibroblast Growth Factor 23 (FGF-23) plays an important role in the regulation of calcium-phosphate-vitamin D axis in CKD. Thus, new insights into the pathogenesis of SHPT give the possibility to have different treatments of this condition in CKD.

SHPT causes both bone (osteitis fibrosa) and extra-skeletal disease (cardiovascular and soft-tissue calcifications, altered erythropoiesis, endocrine dysfunctions) (1). Clinical targets for serum phosphate (P), calcium (Ca), and parathyroid hormone (PTH) levels have been elucidated by the Kidney Dialysis Outcomes Quality Initiative (K/DOQI) Working Group (2). Since alterations in mineral and bone metabolism have classically been named renal osteodystrophy and classified based on bone biopsy, recently, KDIGO (Kidney Disease: Improving Global Outcomes) proposed a new definition for this disease: the CKD-Mineral and Bone Disorder (CKD-MBD) (3). In addition to hypocalcemia and hyperphosphatemia, CKD-MBD is characterized by abnormalities of bone volume, turnover, mineralization, and growth as well as vascular calcification (VC). In fact, VC seems to link abnormalities in serum Ca and P levels and the enhanced mortality risk associated with CKD-MBD. Impressively, VC starts early in CKD and patients with CKD-3, -4 and -5 not undergoing hemodialysis present a significant amount of calcium deposition in the coronary arteries (40-80%). 

During the last decade, many efforts have been made to try to decrease VC progression. The control of serum PTH levels and calcium- and phosphate-load, using aluminum- and calcium phosphate binders, more selective vitamin D receptors activators, and cinacalcet clearly represent our current pharmacological tools to improve quality of life and reduce mortality in CKD (4).

In their manuscript, Rostami et al. (5) showed quality of life in dialysis patients affected by CKD-MBD in Iran. Interestingly, quality of like associated with serum Ca levels, serum calcium-phosphate product levels, and serum PTH levels. Furthermore, hospitalizations were associated with serum P levels. Thus, management of CKD-MBD included phosphate binders (both calcium-based and calcium-free), vitamin D, and cinacalcet, even if treatment of this dramatic complication of CKD remains suboptimal for most patients.
